# A serological and bacteriological survey of brucellosis in wild boar (*Sus scrofa*) in Belgium

**DOI:** 10.1186/1746-6148-8-80

**Published:** 2012-06-18

**Authors:** Fabien Grégoire, Bénédicte Mousset, David Hanrez, Charles Michaux, Karl Walravens, Annick Linden

**Affiliations:** 1 Surveillance Network of Wildlife Diseases, Department of Infectious and Parasitic Diseases, Faculty of Veterinary Medicine, University of Liège, 4000 Liège, Belgium; 2 Unit of Bioinformatics, Department of Animal Sciences, Faculty of Veterinary Medicine, University of Liège, 4000, Liège, Belgium; 3 Department of Bacteriology and Immunology, Veterinary and Agrochemical Research Centre (VAR-CODA-CERVA), 1180, Brussels, Belgium

## Abstract

**Background:**

Brucellosis is frequently reported among wild boar populations in Europe. The aim of the study was to assess the epidemiological situation in Belgium, regarding the steady increase of wild boar populations over the last decades. Several serological tests were used and compared with culture and IS*711* polymerase chain reaction (PCR), to determine the most suitable combination of diagnostic tools for conducting a successful prevalence study in wildlife.

**Results:**

An indirect enzyme-linked immunosorbent assay (iELISA) was used on 1168 sera from hunter-killed wild boar sampled between 2003 and 2007 in 4 natural regions of southern Belgium. Results gave an apparent prevalence of 54.88% (95% CI 52.03-57.73). Prevalence was significantly affected by age and by the year of study, but not by sex nor by the region of sampling. The relative sensitivities of the complement fixation test (CFT), the Rose Bengal test (RBT), and the slow agglutination test (SAT) *versus* the iELISA differed widely between tests, reaching 62.67%, 46.68%, and 34.77%, respectively. The relative specificities of the CFT, RBT and SAT *versus* the iELISA were respectively 99.01%, 92.49%, and 99.1%. From seropositive animals (iELISA), 9% were positive by culture and 24% by PCR when testing spleen and/or tonsils. Sensitivity of the PCR was higher on tonsils than on spleen. All bacterial isolates were identified as *Brucella suis* biovar 2.

**Conclusions:**

Brucellosis is widespread among wild boar in southern Belgium, with seroprevalences having increased over ten years, and constitutes a growing risk of spillback to outdoor-farmed pig herds. The iELISA showed a better sensitivity than the CFT, RBT and SAT. Serological tests must be associated with direct diagnosis and PCR proved more sensitive than culture under wildlife sampling conditions. Spleen and tonsils are lymphoid tissues usually sampled in multi-disease monitoring programs. They remain top-grade organs for direct diagnosis of brucellosis, with a preference for tonsils.

## Background

Brucellosis has not been reported in domestic pigs in Belgium since 1969 [1], but in 1994, *Brucella suis* biovar 2 strains were isolated from hunter-killed boar [2], demonstrating the circulation of the bacteria amongst wild boar *(Sus scrofa)* populations in Belgium. Since then, *B. suis* biovar 2 has been isolated from wild boar in many countries of Central and Western Europe such as France [3], Switzerland [4,5], Germany [6], Spain [7,8], and Croatia [9].

In Belgium as in other European countries, a steady increase of wild boar populations has been observed over the last twenty years. From 1987 to 2007, according to the official census of the Department of Nature and Forestry, the estimated hunting bags of wild boar in southern Belgium (16,844 km^2^) increased from 6,000 to 22,000 [10]. The overabundance of wildlife, recognized as a relevant risk factor for disease transmission between wildlife and domestic animals [11], compromises the health surveillance programs carried out in livestock.

In the domestic pig, brucellosis manifests as infertility and abortions in sows and orchitis in males. Extragenital lesions such as lymphadenitis, subcutaneous abscesses, arthritis, and spondylitis are also common [12]. In wild boar, *B. suis* biovar 2 is often isolated in the absence of gross lesions [13]. Recently, several outbreaks of brucellosis affecting outdoor herds of domestic pigs occurred in Germany and France [3,6], and wild boar appeared as the source of infection. In France, the birth of pig/wild boar hybrids confirmed the intrusion of boar, attracted by sows in heat.

Besides *B. suis* biovar 2, brucellosis in suidae is caused also by *B. suis* biovars 1 and 3. The first is present mainly in North and South America, Asia, and Oceania, while the second is reported in China, the United States, and recently, Europe [12,14].

Unlike *B. suis* biovars 1 and 3, biovar 2 is only weakly zoonotic [12]. Few cases of human brucellosis with isolation of *B. suis* biovar 2 have been reported so far, and they concerned immunocompromised people. In France, for instance, the bacterium was isolated from a hemoculture obtained from a hunter suffering from diabetes and silicosis [15]. Yet the recent isolation of *B. suis* biovar 3 from pigs, wild boar, and horses in a confined region of Croatia shows the emergence of zoonotic biovars in Europe [14,16]. Translocation of wild boar for breeding or hunting purposes increases the risk of spreading of zoonotic brucellosis throughout Europe.

The aims of this study were to determine the apparent seroprevalence of brucellosis in wild boar sampled in the four main hunting regions of Belgium and to identify potential risk factors associated with seropositivity. Direct and indirect brucellosis tests were also compared to determine the most suitable combination of diagnostic tools for conducting a successful prevalence study in wildlife.

## Results

### Serology

Overall, 641 out of 1168 serum samples were considered positive with the indirect enzyme-linked immunosorbent assay (iELISA), giving an apparent seroprevalence rate of 54.88% (95% CI 52.03-57.73). In Table1, the results of the complement fixation test (CFT), the Rose Bengal test (RBT), and the slow agglutination test (SAT) are compared with the indirect enzyme-linked immunosorbent assay (iELISA) data.

**Table 1:** **Results of the serological tests (CFT, RBT and SAT *versus *iELISA) used for diagnosis of brucellosis in wild boar**

**ELISA**		**CFT**		**RBT**		**SAT**	
**Nt**	1168						
**Np**	641	**Nt**	521	**Nt**	532	**Nt**	540
		**Np**	314	**Np**	239	**Np**	186
		**Nn**	187	**Nn**	273	**Nn**	349
		**Nni**	20	**Nni**	20	**Nni**	5
		**Se r**	62.67%	**Se r**	46.68% (42.36-51.00)	**Se r**	34.77% (30.73-38.80)
			(58.44-66.91)		(42.36-51.00)		(30.73-38.80)
**Nn**	527	**Nt**	218	**Nt**	220	**Nt**	227
		**Np**	2	**Np**	16	**Np**	2
		**Nn**	201	**Nn**	197	**Nn**	222
		**Nni**	15	**Nni**	7	**Nni**	3
		**Sp r**	99.01% (97.66-100.00)	**Sp r**	92.49% (88.95-96.03)	**Sp r**	99.11% (97.88-100.00)
			(97.66-100.00)		(88.95-96.03)		(97.88-100.00)

Nt: number of tested sera, Np: number of positive sera, Nn: number of negative sera, Nni: number of non interpretable results.

Se r: relative sensitivity; Sp r: relative specificity. 95% CI are specified.

In the CFT, 4.4% of the samples yielded uninterpretable result, because of either hemolysis or anticomplementary activity. The percentage of unusable samples in the RBT reached 3.7% (hemolysis or the presence of fatty corpuscles affecting the reading). Only 1% of the sera could not be tested by SAT.

Of the samples giving a negative result in the iELISA (n = 527), 20 showed a positive result in one of the other three tests. Two samples showed positivity in the CFT, but the level of positivity was low (it did not exceed 40 I.U./ml). In the RBT, 16 samples gave positive results, with 15 showing a weak reaction (1 +/2 +) and 1 a strong reaction (4 +). In the SAT, 2 sera reacted positively, with a titer superior to 100 I.U./ml.

**Figure 1:**
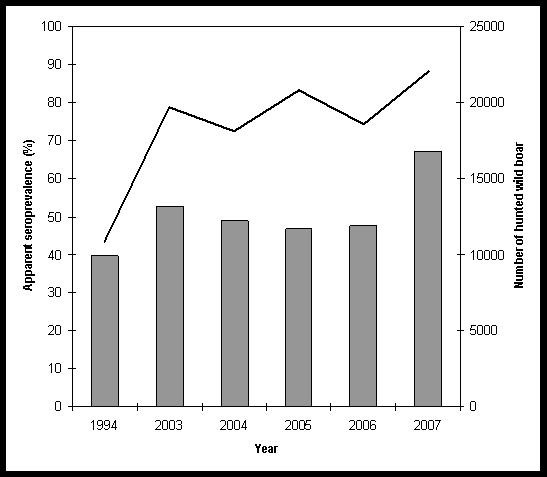
**Apparent seroprevalences (iELISA) towards *Brucella *spp. per year of sampling.** The bar graphic shows the seroprevalences as measured on the left y-axis. The seroprevalence from the study carried out in 1994 is included [2]. The polygon line represents the number of hunted wild boar per year as measured on the right y-axis.

A significant difference exists between the apparent seroprevalence from 1994 (39.7% with 95% CI 31.64-47.79) [2] and the apparent overall one found in this study (*χ*^2^ = 11.62, *P* < 0.001, df 1). The apparent seroprevalences for each age and sex class are presented in Table2. The apparent seroprevalences by year of sampling, along with the annual hunting bag, are presented in Figure1. 

**Table 2:** **Apparent seroprevalences (iELISA) towards *Brucella *spp. in the different age and gender categories in wild boar**

	**Males**		**Females**		**Unknown sex**		**Total**	
	**Np/Nt**	**% (95% CI)**	**Np/Nt**	**% (95% CI)**	**Np/Nt**	**% (95% CI)**	**Np/Nt**	**% (95% CI)**
>** 2 years**	61/68	**89.71 (82.48 - 96.93)**	54/68	**79.41 (69.80 - 89.02)**	-	**-**	115/136	**84.56 (78.49 - 90.63)**
**1 – 2 years**	68/104	**65.38 (56.24 - 74.53)**	120/164	**73.17 (66.39 - 79.95)**	1/1	**-**	189/269	**70.26 (64.80 - 75.72)**
<** 1 year**	90/257	**35.02 (29.19 - 40.85)**	82/223	**36.77 (30.44 - 43.10)**	2/8	**-**	174/488	**35.66 (31.41 - 39.91)**
**Unknown age**	67/127	**52.76 (44.07 - 61.44)**	84/133	**63.16 (54.96 - 71.36)**	12/15	**-**	163/275	**59.27** (**53.47 - 65.08)**
**Total**	286/556	**51.44 (47.28 – 55.59)**	340/588	**57.82 (53.83 - 61.81)**	15/24	**62.50 (43.13 - 81.87)**	641/1168	**54.88 (52.03 - 57.73)**

CI: confidence interval.

Np: number of positive animals, Nt: number of tested animals.

The multivariate logistic regression model performed on iELISA results (Table3) showed year of sampling and age were significantly associated with boar seropositivity. These effects were both significant according to the Wald chi-square hypothesis tests (*P* < 0.01).

**Table 3:** **Summary results of the multivariate logistic regression analysis of risk factors associated with *Brucella *spp. seropositivity (iELISA) in wild boar**

**Risks factors**	**Category levels**	**Multivariate logistic regression results**	
		**Odds ratio (95% CI)**	**Wald test *P *value**
Age			
	adults	1.00	
	sub-adults	0.10 (0.06-0.17)	<0.01
	juveniles	0.43 (0.25-0.74)	<0.01
Year			
	2007	1.00	
	2006	0.42 (0.27-0.65)	<0.01
	2005	0.44 (0.29-0.68)	<0.01
	2004	0.36 (0.23-0.57)	<0.01
	2003	0.39 (0.25-0.62)	<0.01

Overall data of the model: -2 log likelihood = 1037.14, likelihood ratio chi-square = 183.41 (df 6), *P* < 0.01.

In contrast, sex was not a significant influencing factor (*P* = 0.12). The apparent seroprevalence was affected neither by month of sampling (*P* = 0.95) nor by region of sampling (*P* = 0.80).

All the investigated hunting estates harbored seropositive boar.

### Culture and Polymerase Chain Reaction (PCR)

Spleens and tonsils were tested by culture (n = 381) and PCR (n = 389). In a first step, organs were randomly selected from seropositive (iELISA) boar. Organs from seronegative animals were analyzed in a second step.

For the seropositive animals, results differed according to the test used and the organ targeted. None of the seronegative boar tested positive by culture or PCR. The results are presented in Table4.

**Table 4:** Results of culture and of the PCR assay used on organs for diagnosis of brucellosis in wild boar

	**Seropositive boar (iELISA)**		**Seronegative boar (iELISA)**	
	**Np/Nt**	**Prevalence (95% CI)**	**Np/Nt**	**Prevalence**
**Culture**				
Spleen	16/327	4.89% (2.55 – 7.23)	0/28	0.00%
Tonsils	19/228	8.33% (4.75 – 11.92)	0/31	0.00%
Spleen and/or tonsils	31/343	9.04% (6.00 – 12.27)	0/38	0.00%
**PCR**				
Spleen	19/337	5.63% (3.18 – 8.10)	0/33	0.00%
Tonsils	76/236	32.20% (18.72 – 27.90)	0/30	0.00%
Spleen and/or tonsils	84/351	23.93% (19.47 – 28.40)	0/38	0.00%

Np: number of positive animals, Nt: number of tested animals.

In order to compare culture and PCR thoroughly, Table5 (a contingency table) presents the tonsils (n = 259) and spleens (n = 354) on which culture and PCR were performed in parallel. All but one of the tonsils testing positive by culture also tested positive by PCR, and PCR identified as positive 56 tonsils that were negative by culture. Only 5 out of 16 spleens testing positive by culture were detected by PCR, and 13 spleens testing negative by culture tested positive by PCR.

**Table 5:** Comparison of results of culture and PCR assay used on organs for diagnosis of brucellosis in wild boar

	**Tonsils**			**Spleen**		
	**Culture positive**	**Culture negative**	**Total**	**Culture positive**	**Culture negative**	**Total**
**PCR positive**	18	56	74	5	13	18
**PCR negative**	1	184	185	11	325	336
**Total**	19	240	259	16	338	354

Wild boar testing positive by both iELISA and PCR (n = 84) showed various serological profiles (Table6): 83.33% (75.06-91.60), 62.34% (51.51-73.16), and 60.00% (49.26-70.74) tested positive by CFT, RBT, and SAT respectively.

**Table 6:** Results of the serological tests (CFT, RBT, and SAT) for wild boar showing positive results in the iELISA and PCR assay on organs for diagnosis of brucellosis

	**Not tested**	**Uninterpretable**	**Negative**	**Positive samples titer (I.U./ml) or degree of agglutination**						**Np/Nt (%)**	
**iELISA**	0	0	0	**1.875-3.75**	**3.75-7.5**	**7.5-15**	**15-30**	**30-60**	**>60**	84/84	**(100%)**
				1	6	26	30	12	9		
**CFT**	3	3	13	**20-40**	**40-80**	**80-160**	**160-320**	**> 320**		65/78	**(83.33%)**
				7	12	17	16	13			
**RBT**	5	2	29	**+**	**++**	**+++**	**++++**			48/77	**(62.34%)**
				7	4	30	7				
**SAT**	3	1	32	**30-100**	**100-400**	**> 400**				48/80	**(60.00%)**
				25	19	4					

Np: number of positive animals, Nt: number of tested animals.

All isolates (n = 35) were confirmed as *B. suis* biovar 2 according to routine typing methods. No *Yersinia* sp. was isolated from tonsils (n = 110) by culture.

## Discussion

Over this five-year investigation, 1168 wild boar were tested. The results show that brucellosis is endemic in wild boar in southern Belgium. By comparison with a previous study carried out in the same region with the same iELISA [2], an upward tendency was observed in the present work**.** As expected, the four serological tests showed different results, the highest apparent seroprevalence being observed with the iELISA (54.9%). These differences are due not only to the intrinsic factors of each test but also to the immunoglobulin classes that the tests target [17]. The iELISA detects IgG1 and IgG2 (isotopes detected with protein G). These immunoglobulins are present in the later stage of infection and persist over a long period of time [18]. The CFT and RBT mainly detect IgG1, and the principle of SAT is to detect anti-agglutinin antibodies mainly of the IgM isotype, markers of acute infection. In this study, the large proportion of wild boar showing positive results in the iELISA and a lower seroprevalence in the SAT suggests that brucellosis is chronic in wild boar populations in the investigated area. Godfroid and coworkers report the same trends, with apparent seroprevalences of 39.72% (31.64-47.79) and 0.71% (0.00-2.09) measured by iELISA and SAT respectively [2]. Although the relative sensitivities of the CFT, RBT, and SAT are low compared to that of the iELISA, their relative specificities are above 90%. A few ELISA-negative samples were positive by CFT or RBT, but positive reactions were weak in these tests. On the other hand, both ELISA-negative, SAT-positive samples showed a high titer in the SAT test. These boar might have been sampled at an early stage of the humoral response, before the appearance of IgGs. The lesser sensitivities of the CFT, RBT, and SAT are confirmed by the serological profiles of the PCR/ELISA-positive boar.

The apparent seroprevalences reported in the present study are higher than those mentioned in other European reports. Results obtained with an iELISA were around 35% in the Canton of Jura, Switzerland [4], 22% in northeastern Germany [19], and ranged from 25% to 46% in different regions of Spain [8]. In France, the apparent seroprevalence varied from 20% to 35% according to the department [20], but these results were based on CFT and RBT used in serial or parallel testing. Serological results should be compared with caution because of the different tests used.

The circulation of pathogens within wild populations may be influenced by artificial management, and notably by fencing, translocations, and feeding. The impact of these factors is not fully understood, however. In one study, several pathogens including *Brucella* showed higher prevalences in fenced estates than in open ones [21], but other surveys conducted in Spain [7] and France [22] showed no relationship between the apparent prevalence of brucellosis and wild boar management or density.

In southern Belgium, it is forbidden to fence hunting estates, but artificial feeding has been practiced in recent years as supplementation during the winter, as a dissuasive measure aiming to reduce crop damage by wild boar or as attractive measure during hunting season. Clearly, artificial feeding causes spatial aggregation of wild boar and thus, presumably, increased contacts among animals. This factor, associated with the steady increase in wild boar populations, could be linked to the rise in seroprevalence detected between 1994 and 2007 in Belgium.

Our study, however, shows no significant differences in prevalence between regions, even though inter-regional disparities in wild boar densities are observed. According to the official 2007 census of wild boar populations [10], the Famenne region has the highest density: 68 individuals per 1000 ha, *versus* 39 per 1000 ha in the Condroz. These official data are means for defined regions, and do not take local aggregation into account. The 21 hunting zones investigated in this study were all characterized by high wild boar density, as evidenced by annual hunting bags.

In the Famenne and Ardenne regions, where high wild boar densities overlap with outdoor pig farming, there is an increased risk of disease transmission between wild and domestic suids. A similar situation is reported in Switzerland [5]. Preventive actions should concern both farmers and hunters. Outdoor pig farming requires setting up double fences and maintaining sows indoors during heat. On the other hand, hunting stakeholders must control translocations and the release of farm-bred « wild boar », which is strictly prohibited. Likewise, surveillance strategies for brucellosis must include both pigs (in-depth analysis of reproductive disorders) and wild boar. In the latter, surveillance is all the more important because *Brucella* infection is silent in most cases. As observed previously [4,8], the seroprevalence increases with age and does not differ between females and males.

The seroprevalence was significantly higher in 2007 than in previous years of sampling. The reason for this increase is unknown, but as mentioned above, it could be related to the steady rise in wild boar numbers. Further studies will confirm or not an upward trend.

*Brucella* infections can be diagnosed unequivocally only by isolating and identifying *Brucella* spp. or detecting the bacterial DNA by PCR [17]. The PCR used in this study is known to be highly specific for *Brucella* spp. [23,24]. Isolating the bacteria by culture appears less sensitive than the PCR, since only 1 out of 4 PCR-positive samples yielded a positive culture, whatever the organ. A lower sensitivity of the culture method has been reported in other studies [25,26] and is partly related to wildlife sampling conditions, which do not always guarantee good quality of the sampling, adequate transport, and proper tissue conservation. This suggests that a PCR approach, considered more robust under field conditions, should be preferred in large-scale and long-term wildlife surveys. Accordingly, a field study performed in Switzerland and using IS*711* real-time PCR gave excellent results for detection of *Brucella* spp. infections in wild boar [25].

Whatever the method used in our study, *Brucella* or its DNA was more frequently detected in tonsils than in spleen. The recorded bacteriological prevalences were 8.33% (culture) and 32.20% (PCR) for tonsils *versus* 4.89% and 5.63% for spleen. This could mean that in splenic tissue the concentration of *Brucella* DNA is lower, and often below the detection limit of the test. Real-time PCR assays, having higher sensitivity [25], could increase detection of the bacteria within the selected organs. Another cause of false-negative PCR results might be the presence in the spleen of polymerase inhibitors such as blood constituents [26-28]. Variability in the location and quantity of *Brucella* bacteria among targeted organs has been documented [25] and is likely to correlate with the stage of infection in individual animals.

Between the iELISA and PCR results, discrepancies were observed. The high number of iELISA-positive samples that were PCR-negative is worth stressing. This might reflect a lack of sensitivity of the PCR method, a lack of specificity of the iELISA, or both, to some extent. No “gold-standard” test is available for determining the true serological prevalence towards *Brucella* spp. [17]. The high-sensitive iELISA was used in first step for optimizing the detection of carriers at the expense of specificity. Thus the overall prevalence could be overestimated. Nevertheless the iELISA detected all the PCR-positive boar, unlike the three other tests. Cross-reactions seen with other bacteria are a well-known problem in serological diagnosis of brucellosis [25,29]. Because of a shared O-chain on the lipopolysaccharides (LPS), humoral responses induced by some bacteria and notably *Y. enterocolitica* O:9 (Y09) are indistinguishable in iELISAs from those induced by smooth *Brucella* species. In the present study, although no *Yersinia* sp. was isolated from tonsils, the real impact of Y09 on our serological results remains unknown. Y09 has been found in 2.6% of examined wild boar in Switzerland [29]. Alternatively, the discrepancy between the number of seropositive animals and the number of PCR-positive animals might be due, in part, to past infections, with clearance of the bacteria from the host and persistence of anti-*Brucella* antibodies. This “self-limiting” process has been observed in cattle experimentally infected with *B. abortus* biovar 1 or *B. suis* biovar 2 [30].

All wild boar samples (n = 35) isolated in the present study were identified as *B. suis* biovar 2. These results are in agreement with previous studies carried out on wild boar and hares in Europe [2-9,31]. Yet the isolation of *B. suis* biovar 3 from pigs, wild boar, and horses in Croatia shows the emergence of zoonotic biovars in Europe [14,16]. Translocation of wild boar for breeding or hunting purposes increases the risk of spreading zoonotic brucellosis among neighboring countries. In this context it remains important to biotype *Brucella* strains isolated from wildlife in surveillance programs throughout Europe.

## Conclusions

Our study suggests that brucellosis is widespread among wild boar in southern Belgium. Both wild suid populations and brucellosis prevalences have increased from 1994 to 2007, and this constitutes a growing risk of spillback to outdoor-farmed pig herds. For financial and practical reasons, serological tests are the first tools for use in brucellosis prevalence studies in wildlife, but they must be interpreted with caution and the ELISAs used must be expressly validated for use on wild species. Furthermore, it is strongly recommended to associate them with tools for direct diagnosis. In the present study, PCR proved more sensitive than culture under wildlife sampling conditions. Spleen and tonsils are lymphoid tissues usually sampled in multi-disease monitoring programs. They remain top-grade organs for direct diagnosis of brucellosis, with a preference for tonsils.

## Methods

### Study area and animal sampling procedure

This study was conducted in southern Belgium (Region of Wallonia), which includes five distinct natural regions (Hesbaye, Condroz, Famenne, Ardenne, and Lorraine). Among these regions, one (namely Hesbaye, to the north of the Sambre and Meuse valley) has not been colonized by wild boar and thus excluded from the study. Colonization of the Condroz region is recent.

**Figure 2:**
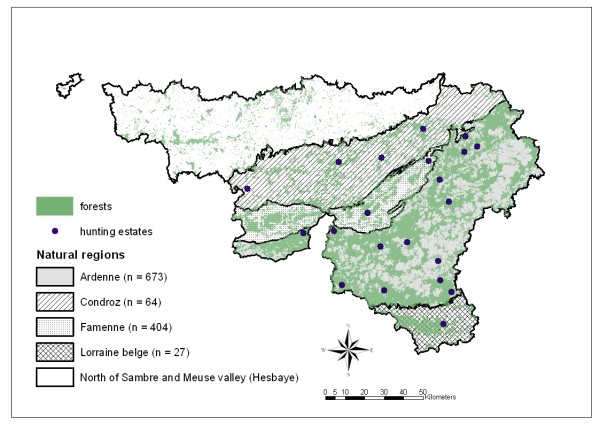
**Geographic distribution of wild boar (n = 1168) sampled from 2003 to 2007 in Wallonia (southern Belgium).** The natural regions are presented with the number of sampled animals (n).

Boar (n = 1168) were investigated in 21 hunting areas spread over the 4 colonized regions (Figure2). The hunting estates investigated were open and characterized by large hunting bags and various degrees of artificial feeding. Blood samples were collected during 5 hunting seasons (October to December, 2003 to 2007).

Individual postmortem examinations included determination of sex and age. Age was determined on the basis of tooth eruption patterns and weight [32]. Animals were classified as juveniles (less than 1 year old), sub-adults (between 12 and 24 months old) and adults (over 2 years old). Numbers of wild boar of each age class are presented in Table2. After examination of the intact whole body, the abdominal, thoracic, and naso-buccal cavities and corresponding organs were checked. Genital organs and joints were checked for gross lesions. Afterwards, blood was immediately collected in dry tubes exclusively by venipuncture from major vessels or by cardiac puncture. Sampling of blood in the thoracic and abdominal cavities was not carried out. Samples were transported to the lab within 12 hours. After centrifugation, sera were stored at −20 °C until analysis. Organ specimens (spleen and palatine tonsils) were sampled over 3 hunting seasons (2005 to 2007) and stored at −20 °C for subsequent analyses.

### Serological tests

All sera were analyzed with an iELISA as described previously [2,33]. Surface LPS from *B. abortus* biovar 1 (strain Weybridge 99) was used as coating antigen and Protein G peroxidase (Biorad, Belgium), which binds to IgG1 and IgG2, as conjugate. The specificity of the iELISA (99.7%) was calculated with 955 negative controls of pig serum [2]. The CFT, RBT, and SAT were performed according to standard procedures [12]. The SAT was performed with adjunction of ethylene diamino tetra-acetic acid (EDTA) in order to inactivate nonspecific IgMs. The iELISA, CFT, and SAT results are expressed in international units (I.U.). In order to compare our iELISA results with those of 1994 [2], a similar cut-off value was used (positive results > 1.875 I.U.).

The cut-off values used for the other tests were: positive results ≥ 30 I.U. and ≥ 20 I.U. respectively for the SAT-EDTA and CFT and any visible reaction of agglutination during the RBT was considered positive.

### Culture and PCR

Prior to bacteriological culture, samples were completely thawed overnight and, before processing, washed with sterile PBS. Ten milliliters of sterile PBS was added to 5 g sample in a disposable bag, which was then sealed and processed in a blender (Stomacher 80^®^).

Farell medium was used for isolating *Brucella* species organisms from spleens and tonsils. Plates were incubated for 7 to 10 days at 37°C in the presence of 7% CO_2_. Suspected or characteristic colonies were checked by Köster staining. All *Brucella*-like cultures were biotyped according to reference typing methods [34].

In parallel with *Brucella* culture, for 110 wild boar sampled in 2005 and 2006, tonsil homogenate was smeared onto *Yersinia* CIN medium, without preliminary enrichment, and incubated for 2 to 3 days at 30°C. Putative *Yersinia* colonies were checked: any oxidase-negative, urease- and catalase-positive colony was identified with the test kit API^®^ 20E (Biomérieux).

In a second step, processed samples were subjected to a *Brucella*-specific PCR assay designed by Ouahrani-Bettache and colleagues [24]. DNA was extracted from tissue with the High Pure PCR Template Preparation Kit (Roche Diagnostics, Mannheim, Germany) according to the manufacturer’s instructions. The oligonucleotide primers were designed from the nucleotide sequence of the multi-copy element IS*711*, specific to *Brucella* spp. These primers were: IS*711* 3’ (5’-GATAGAAGGCTTGAAGCTTGCGGAC-3’) and IS*711* 5’ (5’-ACGCCGGTGTATGGGAAAGGCTTTT-3’).

### Statistical analysis

Statistical analyses were performed using logistic regression (SAS, 1989). Animals were coded as being positive or negative to iELISA and the effect of age, sex, region, month and year of sampling on serological status was investigated. The monthly data were aggregated over the years for statistical analysis. Each explanatory variable was explored in an univariate logistic regression model to test its entry eligibility. The stepwise method with the likelihood ratio test was used to select the independent variables in the multivariate model. The significance level of the score chi-square for variable entry was set to *P* = 0.05. Difference in apparent prevalences between our study and the one carried out in 1994 [2] was tested using chi-square test. Statistical significance in this study was defined at the *P* ≤ 0.05 level.

## Authors’ contributions

FG performed the field work, collected the samples of the study, analyzed the data and drafted the manuscript. BM carried out the laboratory work. DH participated in the field sampling. CM performed the statistical analysis of the data. KW designed the study and supervised the laboratory work. AL was the project leader, supervised the study and contributed to the draft. All authors read and approved the final manuscript.
